# Distinct features of two lipid droplets types in cell nuclei from patients with liver diseases

**DOI:** 10.1038/s41598-023-33977-4

**Published:** 2023-04-26

**Authors:** Norihiro Imai, Yuki Ohsaki, Jinglei Cheng, Jingjing Zhang, Fumitaka Mizuno, Taku Tanaka, Shinya Yokoyama, Kenta Yamamoto, Takanori Ito, Yoji Ishizu, Takashi Honda, Masatoshi Ishigami, Hiroaki Wake, Hiroki Kawashima

**Affiliations:** 1 Department of Gastroenterology and Hepatology, Nagoya University Graduate School of Medicine, 65 Tsurumai-cho, Showa-ku, Nagoya, Aichi 466-8550 Japan; 2 Department of Anatomy (I), Sapporo Medical University, S1W17 Chuo-ku, Sapporo, Hokkaido 060-8556 Japan; 3 Department of Anatomy and Molecular Cell Biology, Nagoya University Graduate School of Medicine, Nagoya, Aichi Japan; 4 Department of Emergency and Critical Care Medicine, Nagoya University Graduate School of Medicine, Nagoya, Aichi Japan

**Keywords:** Hepatology, Hepatitis, Liver, Liver diseases

## Abstract

Lipid droplets (LDs) have been observed in the nuclei of hepatocytes; however, their significance in liver disease remains unresolved. Our purpose was to explore the pathophysiological features of intranuclear LDs in liver diseases. We included 80 patients who underwent liver biopsies; the specimens were dissected and fixed for electron microscopy analysis. Depending on the presence of adjacent cytoplasmic invagination of the nuclear membrane, LDs in the nuclei were classified into two types: nucleoplasmic LDs (nLDs) and cytoplasmic LD invagination with nucleoplasmic reticulum (cLDs in NR). nLDs were found in 69% liver samples and cLDs in NR were found in 32%; no correlation was observed between the frequencies of the two LD types. nLDs were frequently found in hepatocytes of patients with nonalcoholic steatohepatitis, whereas cLDs in NR were absent from the livers of such patients. Further, cLDs in NR were often found in hepatocytes of patients with lower plasma cholesterol level. This indicates that nLDs do not directly reflect cytoplasmic lipid accumulation and that formation of cLDs in NR is inversely correlated to the secretion of very low-density lipoproteins. Positive correlations were found between the frequencies of nLDs and endoplasmic reticulum (ER) luminal expansion, suggesting that nLDs are formed in the nucleus upon ER stress. This study unveiled the presence of two distinct nuclear LDs in various liver diseases.

## Introduction

The nucleus is a membrane-enclosed compartment found in eukaryotic cells, where the chromatin is organized, folded, decoded, and duplicated. This organelle is separated from the cytoplasm by the nuclear envelope (NE), which consists of two phospholipid bilayers: the outer nuclear membrane (ONM) facing the cytoplasm, and the inner nuclear membrane (INM) connecting to the nuclear matrix. The luminal space between the ONM and INM is in continuity with the endoplasmic reticulum (ER) lumen. The NE membrane can form invaginations extending toward the nucleoplasm, called the nucleoplasmic reticulum (NR)^1^, which can be of two types: type 1 NR, which is an extension of the INM; and type 2 NR, which consists of the invagination of both the INM and ONM coupled with cytoplasmic structures^2^. The putative roles of these nuclear structures in the context of disease are currently unknown.

Many different cell types, both eukaryotic and prokaryotic, contain lipid droplets (LDs), which consist of a phospholipid monolayer encasing a core of neutral lipids (triacylglycerols and cholesterol esters, in mammalian cells). In eukaryotic cells, LDs are formed from the ER membrane, where enzymes catalyzing the last step of neutral lipid metabolism reside. Lipid esters are first synthesized and can exist in the phospholipid bilayer to a certain extent. After their content exceeds the solubility limit, they oil out of the bilayer and are sequestered between the two leaflets of the ER membrane, forming a lens-like structure. Finally, lipid esters are released to the cytoplasm, encased in a phospholipid monolayer derived from the ER membrane. After their release to the cytoplasm, the surface of cytoplasmic LDs contacts the ER membrane, allowing lipids and proteins to be bidirectionally exchanged^3–5^. LDs in the cytoplasm store neutral lipids and primarily serve as lipid reservoirs for energy production or membrane synthesis after sequential lipolysis. LDs can also store lipids that are in excess or toxic, preventing lipotoxicity in cells^6^. LDs function as discrete pools of multiple molecule types such as histones^7^, transcription factors^8^, or nucleoporins^9^, thereby regulating their temporal and spatial availability. LDs also contact other organelle membranes, and through these contacts, they are involved in various functions in lipid synthesis, autophagic and proteasomal proteolysis, and viral reproduction, among others^4,5,10^.

In addition to cytoplasmic LDs (cLDs), nuclear LDs have also been reported^11,12^. Nuclear LDs were first visualized in mammalian hepatoma cells using light microscopy^13^ and subsequently in rodent liver cells using electron microscopy^14,15^. The identification of nuclear LDs in mammalian cells has been expanded using modern microscopic techniques, by our group^16–18^ and others^19–23^. Nuclear LDs have been also found in other non-mammalian cells such as budding yeast^24–28^, plant cells^29^, *C. elegans* intestinal cells^30,31^, and fish hepatocytes treated with heavy metals^32^. Nuclear LDs can recruit and activate CDP-choline diacylglycerol phosphotransferase α (CCTα)—a rate-limiting enzyme of the de novo phosphatydilcholine (PC) synthesis pathway^33^—and diacylglycerol acyltransferase and be involved in PC or triglyceride synthesis in hepatoma cells and osteosarcoma cells^18,21,34^. In yeast, nucleoplasmic LDs (nLDs) can regulate transcription of genes related to lipid metabolism by inhibiting the activity of the transcription suppressor Opi1^26,27^. In mammalian cells, nuclear LDs have been found to colocalize with promyelocytic leukemia (PML) bodies^16,21^, which are nuclear structures involved in the regulation of gene transcription^35^. In astrocytoma cells, fatty acid-binding protein 7 and oleic acids can accelerate the formation of nuclear LD–PML body complexes to upregulate the expression level of genes related to tumor proliferation^36^. Despite these important findings, the pathophysiological significance and frequency of nuclear LDs in hepatocytes in liver diseases remains unknown.

In this study, we aimed to analyze the frequency and morphology of nuclear LDs in hepatocytes and elucidate their attributes, which may be involved in their pathophysiological significance in various liver diseases. We found that nLDs accumulated in hepatocytes derived from patients with liver disorders upon ER stress. Conversely, cLDs trapped in type 2 NR correlated with lipid-restricted hepatocytes. The findings in this study provide a basis for further developments in nuclear biology research.

## Results

### Distinct nuclear LDs characterized in liver biopsies

Patient characteristics are summarized in Table 1. Of the 80 patients, 35 were males and 45 were females. Their mean age was 58 years (range: 24 to 89 years). Liver biopsy-proven liver diseases included nonalcoholic steatohepatitis (NASH) in 12 patients, drug-induced liver injury (DILI) in 11 patients, malignant tumor in 22 patients, autoimmune hepatitis (AIH) in 7 patients, and other diseases in 28 patients. Liver biopsies were performed in all cases due to suspicion of liver damage or liver tumor, however, pathological findings revealed "normal liver" in four cases. According to the METAVIR classification of liver fibrosis, the patients had the following scores: 39 patients with F0, 26 with F1, 3 with F2, and 5 with F3. Hematoxylin and eosin (H&E) staining revealed that the frequency of liver steatosis was < 5% in 46 patients, 5–33% in 21 patients, 33–66% in 4 patients, and > 66% in two patients. Blood profiling of the patients is summarized in Table 2. Although the levels of liver enzymes, alkaline phosphatase, and gamma-glutamyl transpeptidase were elevated, those of plasma lipids were within normal range.

**Table 1:** Patients’ characteristics.

Characteristics	Number of patients	nLD (%nuclei)	cLD in NR (%nuclei)
Total patients	80	2.65	0.35
Males	35	2.37	0.39
Females	45	2.87	0.32
Age			
Mean	58		
Range	24–89		
Liver biopsy proven diagnosis			
Non-alcoholic steatohepatitis	12	2.62	0.00
Drug induced liver injury	11	4.48	0.45
Autoimmune hepatitis	7	6.09	0.90
Primary biliary cholangitis	1	1.54	0.00
Alcoholic hepatitis	1	1.48	0.74
Simple steatosis	2	1.53	0.32
HBV hepatitis	3	2.64	0.19
HCV hepatitis	1	3.33	0.00
Sarcoidosis	1	0.00	0.00
Non-specific hepatitis	11	1.46	0.35
Normal liver	4	1.39	0.41
Malignant tumors	22	1.96	0.44
Other tumors	4	0.00	0.00
Liver fibrosis (Metavir)			
F0	39	1.88	0.22
F1	26	4.21	0.58
F2	3	2.55	0.00
F3	5	1.50	0.37
F4	0	–	–
Liver steatosis			
< 5%	46	2.77	0.46
5–33%	21	2.83	0.19
33–66%	4	1.63	0.00
> 66%	2	1.41	0.00

*HBV* hepatitis B virus, *HCV* hepatitis C virus, *nLD* nucleoplasmic lipid droplet, *cLD in NR* cytoplasmic lipid droplet invagination with nucleoplasmic reticulum.

**Table 2:** Blood profile assessment of enrolled patients’.

Laboratory tests	Unit	Mean (range)
Aspartate aminotransferase	U/L	113 (12–1388)
Alanine aminotransferase	U/L	162 (11–2712)
Alkaline phosphatase	U/L	572 (142–8324)
Gamma glutamyl transpeptidase	U/L	164 (11–970)
Triglyceride	mg/dayL	135 (32–647)
Total cholesterol	mg/dL	191 (96–330)
Low-density lipoprotein cholesterol	mg/dL	105 (10–274)
High-density lipoprotein cholesterol	mg/dL	60 (7–103)

**Figure 1:**
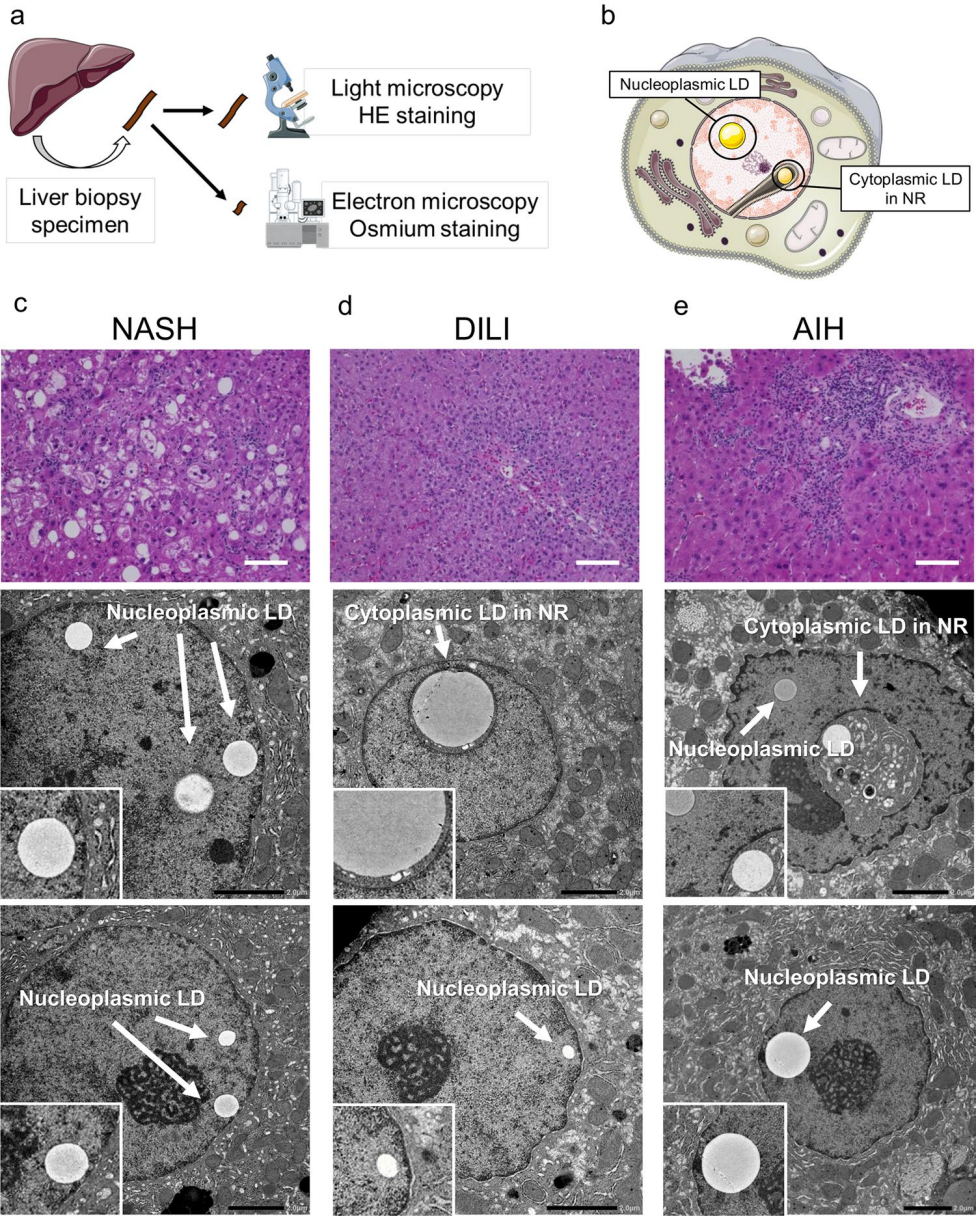
Representative cases of nLDs and cLDs in NR. (**a**) Schematic depiction of the collection of liver biopsy samples and their processing for both hematoxylin and eosin (H&E) staining for light microscopy and osmium staining for electron microscopy. (**b**) Schematic illustration of nLDs and cLDs in NR. (**c–e**) Biopsy sections from patients with (**c**) NASH showing nLDs in hepatocytes and those from patients with (**d**) DILI and (**e**) AIH showing both nLDs and cLDs in NR. *AIH* autoimmune hepatitis, *cLDs in NR* cytoplasmic lipid droplets in the nucleoplasmic reticulum**,** *DILI* drug-induced liver injury, *NASH* nonalcoholic steatohepatitis, *nLDs* nucleoplasmic lipid droplets.

Electron microscopy observations of liver biopsy samples were conducted independently from light microscopy-based clinical diagnosis (Fig. 1a). Depending on the presence of adjacent cytoplasmic invagination of the nuclear membrane, LDs in the nuclei were classified into two types: nLDs and cLDs in NR (Fig. 1b). Six patients were excluded from further analysis because the electron microscopy sample did not contain the corresponding background liver specimen. We observed that 76% of the liver biopsy specimens analyzed presented nuclear LDs in hepatocytes.

### Case assessment toward liver diseases and patient profile

Three representative cases are shown in Fig. 1c–e. The first case is a female in her 70s showing mild increase in aminotransferase levels [aspartate aminotransferase (AST), 125 U/L; alanine aminotransferase (ALT), 106 U/L] and diagnosed as having NASH. Hepatocyte ballooning and mild inflammation were observed in the liver tissue, with 40% steatosis and F2 fibrosis (Fig. 1c). nLDs were found in 5.6% of nuclei, whereas cLDs in NR were not observed (Fig. 1c). The second case is a male in his 20s with elevated AST (211 U/L) and ALT (546 U/L) levels and diagnosed as having DILI. Mild inflammation in the portal area was observed in the liver tissue, with 0% steatosis and F0 fibrosis (Fig. 1d). nLDs were found in 4.2% and cLDs in NR in 1.2% of nuclei in the liver biopsy sample (Fig. 1d). The third case is a male in his 40s with mild elevations in AST (134 U/L) and ALT (250 U/L) levels and diagnosed as having AIH. Severe inflammation in the portal area was observed, with 0% steatosis and F1 fibrosis (Fig. 1e). nLDs were found in 5.7% and cLDs in NR in 5.7% hepatocyte nuclei (Fig. 1e).

**Figure 2:**
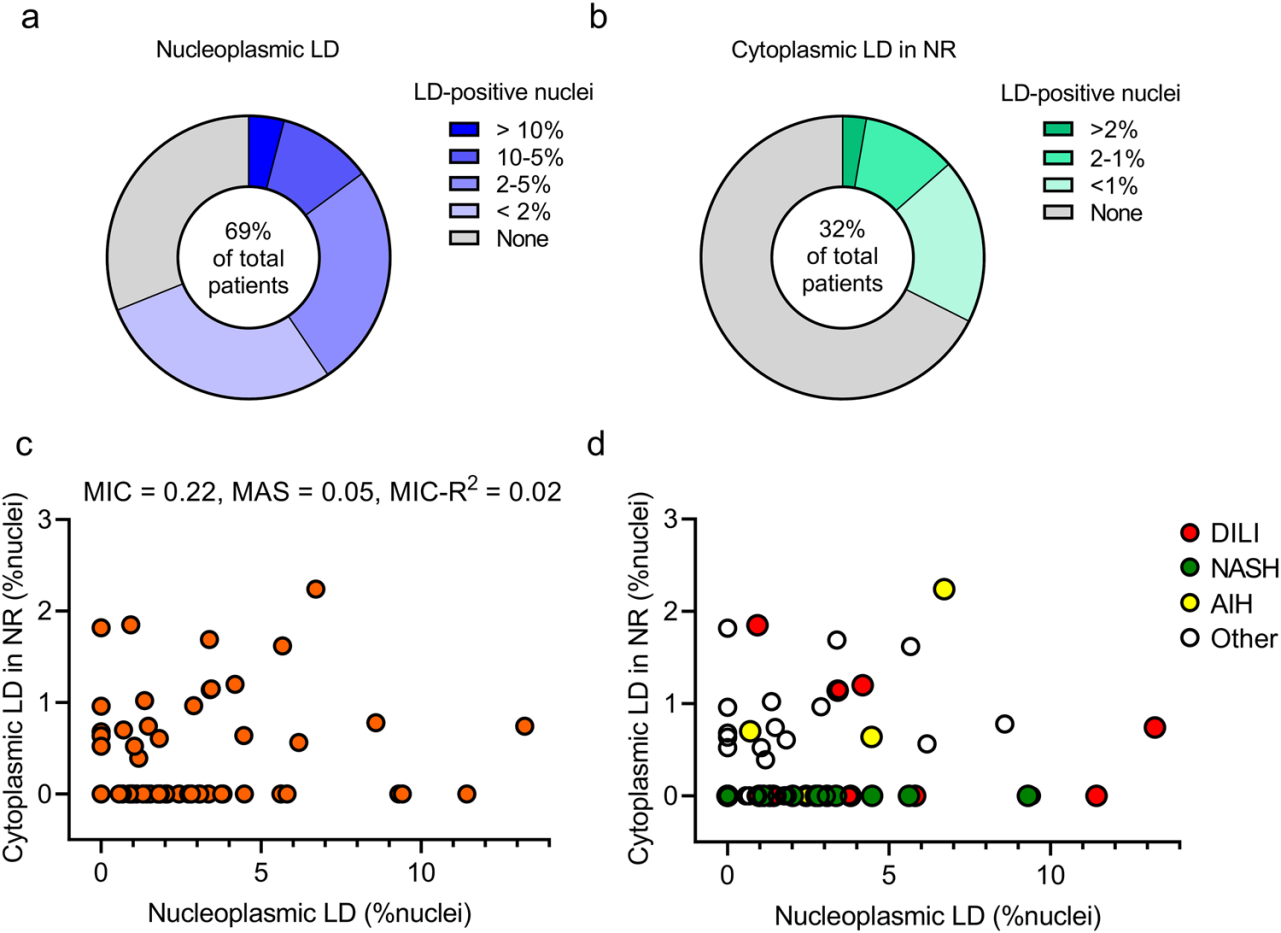
Frequencies and correlations of nLDs and cLDs in NR. The study included 80 patients who underwent liver biopsy. A part of each liver biopsy specimen was dissected and fixed for electron microscopy observation. Six patients were excluded from analysis because the electron microscopy sample did not contain the corresponding background liver specimen. The frequencies of nLDs and cLDs in NR were determined by counting > 100 nuclei per specimen. (**a**) Frequency of nLDs in liver biopsy samples. (**b**) Frequency of cLDs in NR in liver biopsy samples. (**c,d**) Scatter plot showing a correlation between nLD and cLD in NR frequencies. Correlations were analyzed using the MINE method that captures a wide range of associations, functional and otherwise. *AIH* autoimmune hepatitis, *cLDs in NR* cytoplasmic lipid droplets in the nucleoplasmic reticulum**,** *DILI* drug-induced liver injury, *MAS* maximum asymmetry score, *MIC* maximal information coefficient, *MINE* maximal information-based nonparametric exploration, *NASH* nonalcoholic steatohepatitis, *nLDs* nucleoplasmic lipid droplets.

Overall, nLDs were found in 69% of liver biopsy samples, and cLDs in NR were less frequent (32%; Fig. 2a, b). There was no correlation between the frequencies of nLDs and cLDs in NR (Fig. 2c). Although both nLDs and cLDs in NR are most frequently detected in AIH (Table 1), no disease-specific distributions were observed in their frequencies (Fig. 2d). These results revealed that two types of nuclear LDs occur in the human liver.

### Correlation analysis of LDs and clinical parameters

**Figure 3:**
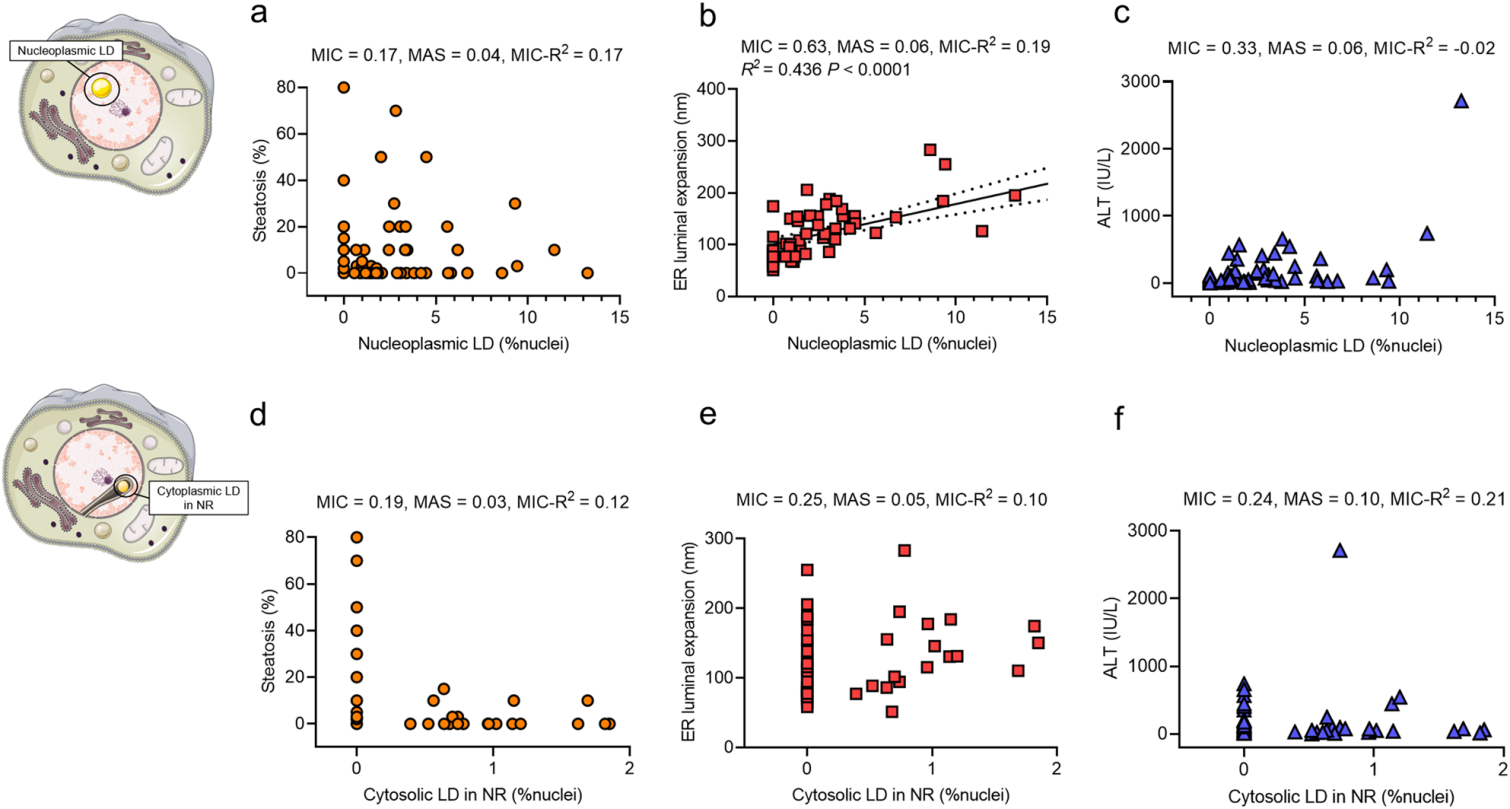
Correlation of nLDs and cLDs in NR with clinical parameters. Correlations were assessed in data from 74 patients who had observable liver specimens in electron microscopy. (**a–c**) Scatter plots showing the correlation between frequencies of nLDs and hepatic steatosis, ER luminal expansion, and plasma level of ALT. Correlations were analyzed by using the MINE method. The black line depicts linear regression; the dotted line shows the 95% confidential intervals. (**d–f**) Scatter plots showing the correlation between frequencies of cLDs in NR and hepatic steatosis, ER luminal expansion, and plasma level of ALT. *ALT* alanine aminotransferase, *cLDs in NR* cytoplasmic lipid droplets in the nucleoplasmic reticulum, *ER* endoplasmic reticulum, *MAS* maximum asymmetry score, *MIC* maximal information coefficient, *MINE* maximal information-based nonparametric exploration, *nLDs* nucleoplasmic lipid droplets.

**Figure 4:**
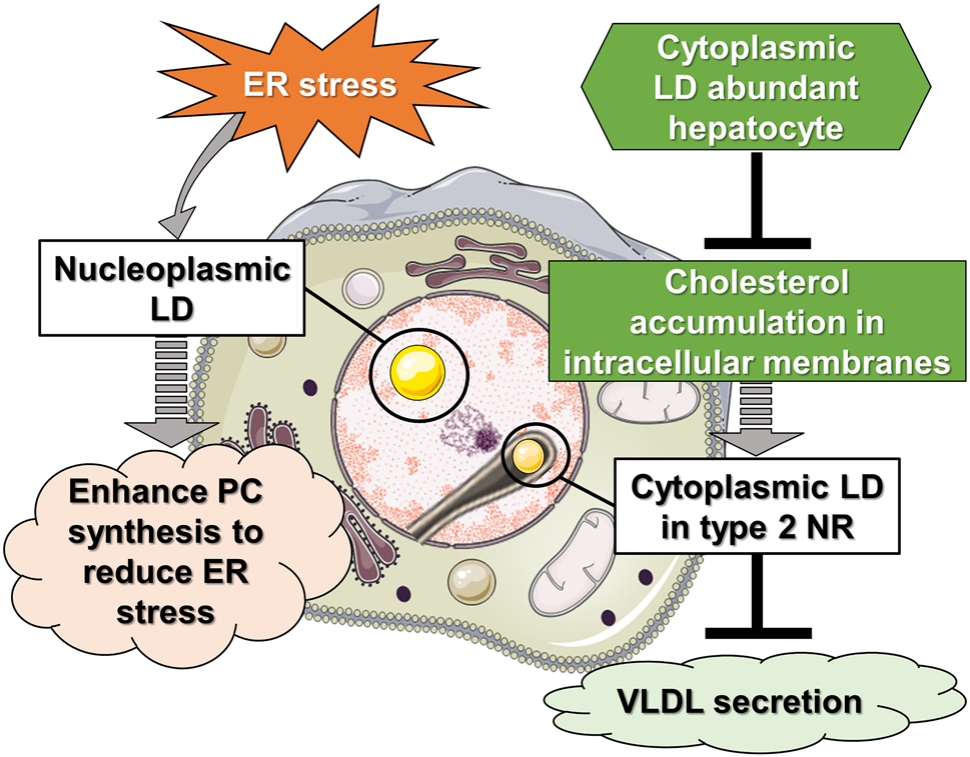
Model depicting two distinct interpretations of nuclear LDs in liver diseases. Schematic representation of potential significance of LDs in the nucleus. Left model: in human hepatocytes under ER stress, secretion of VLDL can be suppressed, leading to accumulation of lipoprotein precursors in the ER lumen. These precursors then translocate into the lumen of type 1 NR (INM invagination), which results in increased amount of nLDs. The surface of nLDs can play a role in induction of PC synthesis to reduce ER stress in various liver diseases. Right model: alternatively, in hepatocytes of patients with low total and LDL cholesterol plasma levels, cholesterols will be sequestered in cellular membranes (e.g. in the NE), which may accelerate the formation type 2 NR (invagination of ONM and INM). cLDs may be initially packed in type 2 NR to be isolated from ER contact sites. This leads to decreased usage of lipids stored in cLDs for VLDL production in the ER. In these circumstances, cLDs in NR may negatively regulate the VLDL secretion machinery in hepatocytes. This machinery may be inhibited when patients’ liver show severe steatosis in which hepatocytes accumulate cholesterols as cholesterol esters in excessive cLDs instead of as free cholesterol in cellular membranes. *cLDs in NR* cytoplasmic lipid droplets in the nucleoplasmic reticulum, *ER* endoplasmic reticulum, *INM* inner nuclear membrane, *LDL* low-density lipoprotein, *nLDs* nucleoplasmic lipid droplets, *NR* nucleoplasmic reticulum, *NE* nuclear envelope, *ONM* outer nuclear membrane, *PC* phosphatydilcholine, *VLDL* very low-density lipoprotein.

Next, we studied the correlations between these two LD types in the nucleus and their clinical characteristics. Although patients who presented with nLDs showed higher levels of liver enzymes in the plasma than those without nLDs, there were no significant differences in tests depending on the presence of nLDs (Table 3). Conversely, patients who presented cLDs in NR showed significantly lower levels of total cholesterol and low-density lipoprotein (LDL) cholesterol, compared to patients without cLDs in NR (Table 3). Thus, the two LD types in the nucleus appeared to be clinically different. Furthermore, nLDs were frequently found in hepatocytes of patients with NASH; however, there was no correlation between the frequency of nLDs and hepatic steatosis, indicating that nLDs do not reflect only cytoplasmic lipid accumulation (Fig. 3a). Positive correlations were found between the frequency of nLDs and ER luminal expansion (Fig. 3b), suggesting nLD formation in the nucleus upon ER stress. Despite the presence of frequent nLDs and severe liver enzyme elevations in two cases, no discernible correlation was observed between the frequency of nLD and liver enzymes (Fig. 3c). Although cLDs in NR also showed no correlation with steatosis, livers with more than 20% steatosis did not show any cLD in NR, indicating that cLD in NR are not formed in hepatocytes with excessive cytoplasmic LDs (Fig. 3d). Furthermore, no correlations were found between the presence of cLDs in NR and ER luminal expansion or liver enzyme (Fig. 3e, f) as well as between these two nuclear LD types and the levels of lipids in the plasma (Supplementary Fig. S1). Based on these results, we speculate two distinct pathophysiological roles of nLDs and cLDs in NR in liver diseases (Fig. 4).

**Table 3:** Comparison of blood profiles depending on the presence of nuclear lipid droplets.

Laboratory tests	Unit	Not present	Present	*P* value
Nucleoplasmic LD				
Aspartate aminotransferase	U/L	52.96	146.2	0.083
Alanine aminotransferase	U/L	52.43	223	0.057
Alkaline phosphatase	U/L	434.2	646.9	0.428
Gamma glutamyl transpeptidase	U/L	144.9	180.7	0.485
Triglyceride	mg/dL	150.4	132.5	0.525
Total cholesterol	mg/dL	195.1	188.5	0.545
Low-density lipoprotein cholesterol	mg/dL	102.4	105.6	0.722
High-density lipoprotein cholesterol	mg/dL	62.05	59.3	0.618
Platelet count	× 10^3^/μL	221.9	218.5	0.883
Cytoplasmic LD in NR				
Aspartate aminotransferase	U/L	96.94	159.5	0.241
Alanine aminotransferase	U/L	132.4	248.2	0.194
Alkaline phosphatase	U/L	644.5	448.1	0.459
Gamma glutamyl transpeptidase	U/L	168.8	171.3	0.961
Triglyceride	mg/dL	137.1	139.1	0.941
Total cholesterol	mg/dL	197.6	176.8	**0.048**
Low-density lipoprotein cholesterol	mg/dL	110.8	92.48	**0.036**
High-density lipoprotein cholesterol	mg/dL	62.64	55.26	0.167
Platelet count	× 10^3^/μL	221.3	216.1	0.819

*LD* lipid droplet, *NR* nucleoplasmic reticulum.

Statistical analyses were conducted using Student’s t-test. Significant values are in bold.

## Discussion

The results obtained in this study revealed that two types of nuclear LDs occur in hepatocytes and uncovered correlations between them and distinct pathophysiological features in human liver disease. To date, there is only one report on the existence of nuclear LDs in human tissues in which two types of nuclear LDs were first identified in hepatocytes from a patient with hepatitis C virus infection by using sequential electron microscopy^23^. Our current findings expand the observations made of nLDs, which occur in the nuclear matrix, and cLDs trapped in type 2 NR, in human hepatocytes of patients with variable clinical backgrounds. In our study, we used electron microscopy to study the ultrastructural components in the nuclei of hepatocytes. This approach unveiled previously unknown characteristics of nuclear LDs based on the presence of adjacent cytoplasmic invagination of the nuclear membrane. Distinguishing these two types of nuclear LDs was only possible with electron microscopy.

Although more detailed analyses are required to identify the functions of both types of nuclear LDs in human hepatocytes, we hypothesize on the physiological significance of LDs in the nucleus (Fig. 4). In hepatocytes, lipids stored in cLDs are released by lipolysis and can be re-used in the ER lumen to generate precursors of very low-density lipoprotein (VLDL)^37^. We previously reported that after VLDL secretion is suppressed under ER stress conditions in hepatoma cells and mouse liver hepatocytes, lipoprotein precursors accumulate in the lumen of the ER and are then transferred into the nucleus through the invagination of the INM, which corresponds to type 1 NR. These luminal LDs derived from lipoprotein precursors are then converted to nLDs in the nucleoplasm after budding from the INM membrane^16,17^. nLDs can recruit and activate CCTα to its surface phospholipid monolayer^17,21^ and can be a platform to synthesize PC, leading to increased membrane synthesis and expansion of the ER volume, which counteracts ER stress^12^. In the present study, we found that hepatocytes with minimal hepatic damage do not present intranuclear LDs, whereas under ER stress involving liver enlargement, 5–10% of hepatocytes show intranuclear LDs. Thus, we hypothesize that nLDs have a role in enhancing PC synthesis to reduce ER stress in various liver diseases (Fig. 4, left model). Conversely, the frequency of cLDs in NR was relatively higher in hepatocytes from patients with lower plasma LDL levels. The function of type 2 NR has been proposed in several reports: nuclear calcium transient in cardiomyocytes^38^; accumulation of mRNA in the surrounding cytoplasmic area and activation of protein translation^39^, and a compartment of active PC synthesis in pre-adipocytes and fibroblasts^40^. The specific roles of type 2 NR in hepatocytes are unknown; however, our findings suggest that cLDs may be first sequestered in type 2 NR and separated from the ER, causing limited lipid transfer between the two organelles. This could then result in a decrease in lipoprotein synthesis in the patient’s liver (Fig. 4, right model).

Several proteins have been proposed to regulate NR membrane invaginations; however, it is difficult to discriminate between the two types of membrane invaginations without electron microscopy imaging. Lamins, CCTα,^41,42^ and the INM protein emerin^38^ have been suggested as positive regulators. PML isoform II can facilitate the formation of nLDs as well as type 1 NR by overcoming the effects of negative NR regulators such as Reeps (ER shaping proteins)^43^ and Suns (INM proteins connecting cytoplasmic/nucleoplasmic skeleton fibers)^16,44^. Moreover, in yeast, the increased amount of cellular sterols and accumulation of phosphatidic acids in the nuclear membrane can accelerate the invagination of the NE while trapping cytoplasmic materials, as these lipids tend to induce negative curvature of the membrane^45^. In our study, patients with low levels of sterols (total cholesterol and LDL-cholesterol) in the plasma showed relatively more cLDs trapped in type 2 NR, suggesting that sterols are highly sequestered in cellular membranes (e.g. nuclear membrane), which may initiate invaginations of both the ONM and INM in hepatocytes. Our study also showed that cLDs in NR are fewer in hepatocytes derived from patients’ liver with more than 20% steatosis. In such cLD-abundant hepatocytes, cholesterols will be accumulated as cholesterol esters in cLDs rather than as free cholesterol in cellular membranes, which may inhibit the formation of type 2 NR-trapped cLDs (Fig. 4, right model). Furthermore, depletion of the ER transmembrane protein Seipin and its homologs tends to increase the amount of intranuclear LDs in yeast^24,25^, plant cells^29^, intestinal and germ cells in *C. elegans*^30^, and human osteosarcoma cells^18^. In mammalian cells, seipin seems to act as a switching molecule to determine cytoplasmic/nucleoplasmic LD formation likely by regulating the translocation of lipid-synthesizing enzymes from the ONM to the INM, where lipid esters are synthesized to form nLDs^12,18^. Such direct nLD formation machinery could be present in not only non-hepatic cells but also hepatocytes, in addition to the type 1 NR-mediated mechanism. Further studies are required to identify the expression patterns of such molecules mentioned above in patients with liver diseases, which will clarify the molecular mechanisms that induce the formation of nuclear LDs and invagination of nuclear membranes.

Even in the limited number of studied cases, it is evident that nLDs are abundant in patients with NASH and DILI and cLDs in NR in patients with DILI and AIH. Although nLDs were frequently found in hepatocytes of patients with NASH, no cLDs in NR were observed. Further studies are warranted to elucidate the pathophysiological significance of nLDs and cLDs in NR in different diseases. As we had included only diseased livers, additional studies are needed on healthy liver tissue, to discriminate between putative physiological roles of these droplets.

In summary, we explored the pathophysiological features of intranuclear LDs in liver diseases and found that nLDs were frequently observed in hepatocytes of patients with AIH, DILI, and NASH. In contrast, cLDs in NR were not observed in NASH liver, suggesting that formation of cLDs in NR is inhibited in cLDs-abundant hepatocytes. Together, our results unveiled the presence of nuclear LDs in human hepatocytes and their correlation with the nuclear ultrastructure underlying pathological conditions.

## Methods

### Informed consent

Written informed consent for sample collection was obtained before the biopsy. This study was conducted in accordance with the principles of the Declaration of Helsinki and was approved by the Bioethics Review Committee of Nagoya University Hospital (2020-0229).

### Human liver biopsy sample

Eighty patients who underwent liver biopsies for liver disease diagnosis at the Nagoya University Hospital (Nagoya, Aichi, Japan) were included in this study (Fig. 1a). Liver biopsy procedures were performed percutaneously using an ultrasound imaging guide. Briefly, disinfectant was applied below the right rib cage, and a small incision was made after application of a local anesthetic. A biopsy needle (16G Bard Monopty Biopsy Gun, Bard Peripheral Vascular, Inc., Tempe, AZ, USA) was inserted into the right lobe of the liver. A part of the liver biopsy specimen (approximately 1 mm^3^) was dissected using a scalpel and fixed in 0.1 M cacodylate buffer containing 2.5% glutaraldehyde. In cases of biopsies aiming at liver tumor diagnosis, a portion of surrounding normal liver tissue was also collected for electron microscopy. A mixture of 1% osmium tetroxide and 0.1% potassium ferrocyanide in 0.1 M sodium cacodylate buffer was added post-fixation. The samples were embedded in epoxy resin, and ultrathin sections were imaged using a JEOL JEM-1400PLUS electron microscope (JEOL Ltd., Tokyo, Japan) operated at 100 kV. Digital images were captured using an EM-14661FLASH camera (JEOL Ltd.). LDs in the nucleus were classified into two types, depending on the presence of adjacent cytoplasmic invagination with nuclear membrane: nLDs and cLD invagination with NR (cLDs in NR) (Fig. 1b)^2^.

### Histological assessment

Histological assessment of hepatocyte ultrastructures was performed independently in the absence of clinical information. The frequencies of nLDs and cLDs in NR were determined by counting more than 100 nuclei per specimen. For each specimen, a single section was used for electron microscopy analysis. Additional sections were used in cases of insufficient fields of view or when the number of hepatocytes was low. The maximum vertical diameter of the rough ER close to the nucleus (for areas of the ER less than 2.0 µm from the ONM) was measured at three points from three random images, and the average value determined. The vertical diameter was defined as the diameter of the tubules that are perpendicular to the plane of the section. The METAVIR scoring system was used to assess fibrosis and inflammation, which is a widely used scoring system for the histological assessment of liver disease, specifically for the classification of chronic liver disease and its severity^46^. Liver steatosis was scored based on the nonalcoholic fatty liver disease (NAFLD) Activity Score, which is a semi-quantitative scoring system for the assessment of liver histology in patients with NAFLD^47^. This grading is based on the visual analysis of liver biopsy specimens and the assessment of the amount of lipid accumulation in hepatocytes.

### Clinical tests

Blood samples were collected one day before liver biopsies. All laboratory tests were performed at Nagoya University Hospital. Liver biopsy samples were fixed and embedded in paraffin for H&E staining. Histological diagnosis was made by certificated pathologists.

### Statistical analysis

Correlations were analyzed using the maximal information-based nonparametric exploration (MINE) method^48^, which captures a wide range of associations, both functional and otherwise. The MINE statistics provide quantitative evaluations of different aspects of the relationship between two variables. In particular, MINE returns three statistics: maximal information coefficient (MIC), maximum asymmetry score (MAS), and MIC-R^2^. MIC relates to the strength of the relationship and can be interpreted as a correlation measure. The MIC value ranges [0–1], tending to 0 for statistically independent data and approaching 1 in probability for noiseless relationships. MAS represents the deviation from monotonicity. Larger MAS values mean not monotonic samples. MIC-R^2^ is the difference between the MIC value and the Pearson’s correlation coefficient; therefore, larger MIC-R^2^ values correspond to nonlinear regressions.　Correlation coefficients were calculated for those correlations with an MIC value of 0.5 or greater, depending on the relationship type. Student’s *t*-test was used to compare the differences in blood parameters for liver biopsies with and without LDs in the nucleus. Statistical significance was defined at *P* < 0.05.

MINE statistics were calculated with EZR (Saitama Medical Center, Jichi Medical University, Saitama, Japan), which is a graphical user interface for R (The R Foundation for Statistical Computing, Vienna, Austria)^49^.

All graphs were generated using GraphPad Prism version 9 (GraphPad Software, San Diego, CA, USA).

## Supplementary Information

Supplementary Figure S1.

## Data Availability

The data that support the findings of this study are available from corresponding authors on request.

## Abbreviations

AIH: Autoimmune hepatitis
ALT: Alanine aminotransferase
AST: Aspartate aminotransferase
CCTα: CDP-choline diacylglycerol phosphotransferase α
cLD: Cytoplasmic lipid droplet
DILI: Drug-induced liver injury
ER: Endoplasmic reticulum
H&E: Hematoxylin and eosin
INM: Inner nuclear membrane
LD: Lipid droplet
LDL: Low-density lipoprotein
MAS: Maximum asymmetry score
MIC: Maximal information coefficient
MINE: Maximal information-based nonparametric exploration
NAFLD: Nonalcoholic fatty liver disease
NASH: Nonalcoholic steatohepatitis
NE: Nuclear envelope
nLD: Nucleoplasmic lipid droplet
NR: Nucleoplasmic reticulum
ONM: Outer nuclear membrane
PC: Phosphatydilcholine
PML: Promyelocytic leukemia
VLDL: Very low-density lipoprotein
