# Supplementary material for: Distinct features of two lipid droplets types in cell nuclei from patients with liver diseases
Source: Sci Rep. 2023 Apr 26;13:6851. doi: 10.1038/s41598-023-33977-4 (PMC10133345; doi:10.1038/s41598-023-33977-4)
Supplement: Supplementary file 1 — Supplementary Figure S1. [file 41598_2023_33977_MOESM1_ESM.docx]

**Supplementary Information**

Distinct Features of Two Lipid Droplets Types in Cell Nuclei from Patients with Liver Diseases

Norihiro Imai^1^*^#^, Yuki Ohsaki^2^*^#^, Jinglei Cheng^3^, Jingjing Zhang^1^, Fumitaka Mizuno^1^, Taku Tanaka^4^, Shinya Yokoyama^1^, Kenta Yamamoto^1^, Takanori Ito^1^, Yoji Ishizu^1^, Takashi Honda^1^, Masatoshi Ishigami^1^, Hiroaki Wake^3^, Hiroki Kawashima^1^

**
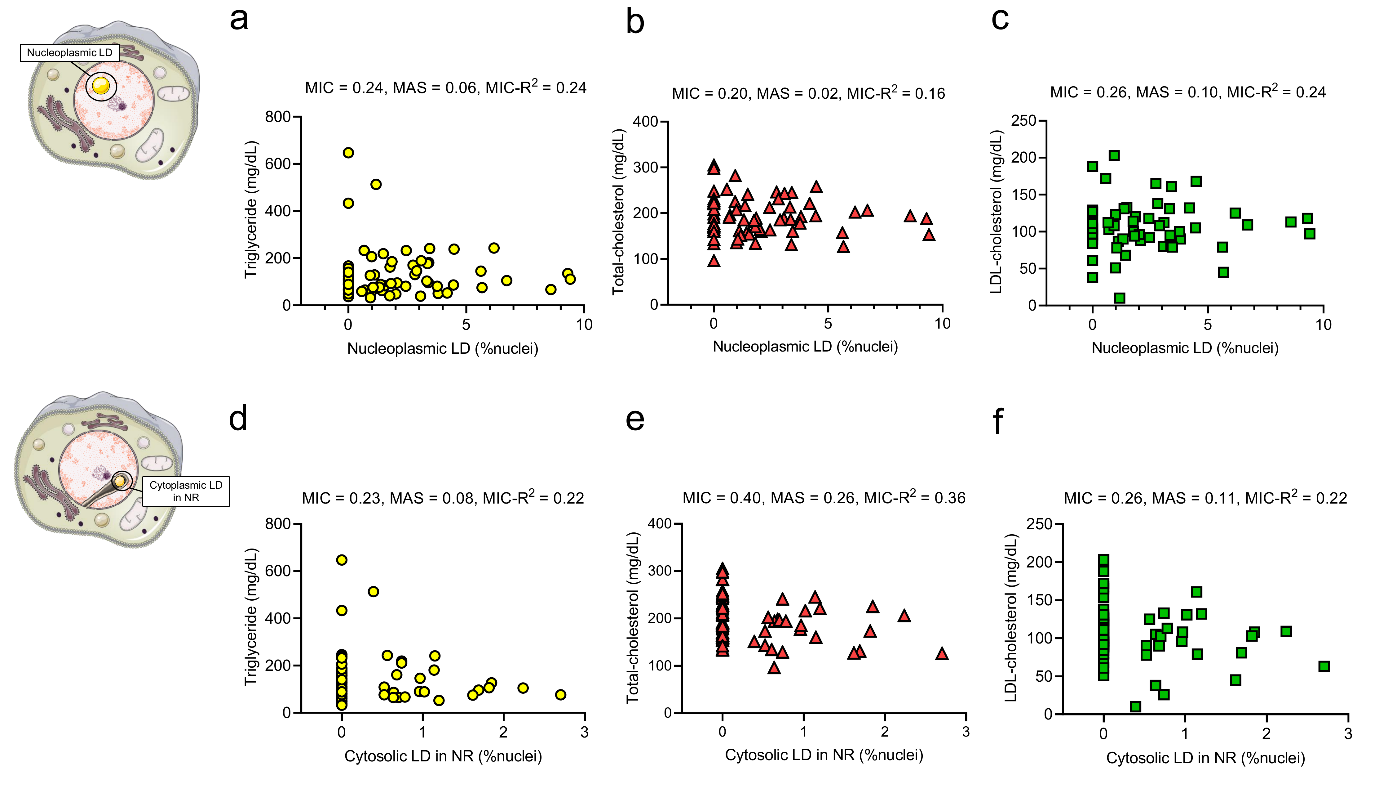
**

**Supplementary Fig. S1. Correlations between nLDs and cLDs in NR and plasma lipid parameters.**

Correlations were assessed in data from 74 patients who had observable liver specimens in electron microscopy. Correlations were analyzed using the MINE method. (a–c) Scatter plots showing the correlation between frequencies of nLDs and triglyceride, total cholesterol, and LDL cholesterol levels in the plasma. (d–f) Scatter plots showing the correlation between frequencies of cLDs in NR and triglyceride, total cholesterol, and LDL cholesterol levels in the plasma.

cLDs in NR, cytoplasmic lipid droplets in the nucleoplasmic reticulum; LDL, low-density lipoprotein; MAS, maximum asymmetry score; MIC, maximal information coefficient; MINE, maximal information-based nonparametric exploration; nLDs, nucleoplasmic lipid droplets.
